# Mitochondrial cardiomyopathies: navigating through different clinical and management pictures between adult and paediatric forms

**DOI:** 10.3389/fcvm.2025.1621096

**Published:** 2025-07-03

**Authors:** Rachele Adorisio, Nicoletta Cantarutti, Barbara Siri, Elisa Bellettini, Gessica Ingrasciotta, Erica Mencarelli, Francesca Graziani, Rosa Lillo, Sara Di Marzio, Corrado Di Mambro, Fabrizio Drago, Antonio Amodeo, Diego Martinelli

**Affiliations:** ^1^ Heart Failure and Transplant, Mechanical Circulatory Support Unit, Bambino Gesù Children’s Hospital, IRCCS, Rome, Italy; ^2^ Pediatric Cardiology and Arrhythmias Unit, Bambino Gesù Children’s Hospital, IRCCS, Rome, Italy; ^3^ Division of Metabolic Diseases and Hepatology, Bambino Gesù Children’s Hospital, IRCCS, Rome, Italy; ^4^ Department of Paediatrics, Città della Salute e della Scienza, OIRM, University of Turin, Turin, Italy; ^5^ Department of Cardiovascular Medicine, Fondazione Policlinico Universitario A. Gemelli IRCCS, Rome, Italy

**Keywords:** mitochondrial disease, heart, cardiac manifestations, cardiomyopathies, children, adults

## Abstract

Mitochondrial diseases (MD) represent a group of rare disease with an estimated prevalence of 5–12 per 100,000 individuals, with a prevalence at birth of 1:5,000 and with childhood-onset of 5–15 per 10,000. They are characterized by a multisystemic phenotype with neurodegenerative, neuromuscular, ophthalmological, endocrinological, gastroenterological and cardiac manifestations. MD can present as a systemic disease or with single organ involvement. When cardiac involvement is the presenting feature, physicians must have a high level of suspicion to search for other organ involvement that can lead to the diagnosis. Cardiovascular manifestations are frequently reported in MD with a significant contribute to mortality. Cardiac involvement is particularly represented in MD with an estimated incidence of 20%–40% in children. Presentation is manifesting as a wide range of cardiac disease, encompassing cardiomyopathy, disturbance of conduction systems, aortopathy and pulmonary hypertension. The aim of this review is to provide a cardiological perspective on the cardiac involvement occurring in the main MD, according to the age of onset, clinical and phenotypic presentation, focusing on the paediatric and adult differences.

## 1 Introduction

Mitochondrial diseases (MD) are a group of rare disorders with an estimated prevalence of 5–12 per 100,000 individuals, with a prevalence at birth of 1:5,000 and with childhood-onset of 5–15 per 10,000 (1, 2). They are characterized by multisystemic involvement, often presenting with neurodegenerative, neuromuscular, ophthalmological, endocrinological, gastrointestinal, and cardiac manifestations (1). Among these, cardiac involvement is particularly common in children, with an estimated incidence of 20%–40%, and significantly contributes to overall morbidity and mortality (3, 4).

Mitochondria are unique organelle, where prokaryotic and eukaryotic mechanisms exist and are capable of fission and fusion, as well as trans-cellular migration. Mitochondria contribute to many processes central to cellular function and dysfunction including calcium signalling, cell growth and differentiation, cell cycle control and cell death. Mitochondrial shape and positioning in cells is crucial and is tightly regulated by processes of fission and fusion, biogenesis and autophagy, ensuring a relatively constant mitochondrial population. Mitochondria are different between tissue, say liver, kidney, brain, heart, and leukocytes. Presence of double membrane necessitates mitochondrial targeted drug delivery mechanisms. Usually, the mitochondrial DNA in the same tissue is similar and considered to be homoplasmic. With accumulating mutations, the same become heterogenous and hence termed heteroplasmic. This leads to a threshold effect, namely, the genetic mutation is confined to some mitochondria, but not to all, and the symptoms manifests once the number of mutated mitochondria exceed a threshold percentage. This threshold level could vary between various tissues. In many subjects increased mitochondrial biogenesis compensates for the defect. Two hit hypotheses involving positively or negatively modifier genes is also postulated for incomplete and organs specific manifestations of the disease Mitochondrial dysfunction is implicated in metabolic and age-related disorders, neurodegenerative diseases and ischemic injury in heart and brain (5).

Cardiac manifestations in MD can vary widely and include cardiomyopathies, conduction system disturbances, aortopathies, and pulmonary hypertension. In pediatric cases, cardiologists are often involved early when cardiac issues are identified within a multisystemic context. In adult patients, MD is rarely considered in the differential diagnosis of cardiac disorders, especially when cardiac symptoms present in isolation. This diagnostic gap contributes to underdiagnosis or delayed diagnosis in adults.

In recent years, mitochondrial dysfunction has gained attention for its role in the pathogenesis of common cardiac conditions, such as heart failure and ischemic heart disease. As the heart is a highly energy-dependent organ, reliant on mitochondrial oxidative phosphorylation (OXPHOS) for approximately 95% of its ATP production, it is particularly vulnerable to mitochondrial defects (4). The resulting energy depletion, along with increased oxidative stress, calcium dysregulation, and apoptosis, can all contribute to myocardial dysfunction and arrhythmogenesis (5, 6).

This review aims to outline the clinical presentation and diagnostic approach to cardiac involvement in mitochondrial diseases, with a focus on both pediatric and adult populations. Additionally, it explores key mitochondrial syndromes associated with cardiovascular complications, offering nsights into their underlying mechanisms and genetic basis.

## 2 Pathophysiology and genetic basis of cardiac involvement in mitochondrial diseases

The heart is a highly energy-dependent organ, relying on OXPHOS for 95% of its ATP production. In MD, impaired mitochondrial function leads to energy depletion, triggering a cascade of cellular dysfunctions. These include disturbed calcium handling, increased reactive oxygen species (ROS), nitric oxide deficiency, and dysregulated apoptosis, all contributing to myocardial damage (4).

Cardiac muscle cells require continuous ATP to support electrical activity and contractility. Reduced ATP and increased ROS make cardiomyocytes more vulnerable to dysfunction and arrhythmias (6). Mitochondrial dysfunction also alters the function of critical ion channels sodium, potassium, and gap junction protein connexin 43, leading to electrical instability. Specific abnormalities include increased late sodium current, reduced potassium current, and impaired calcium homeostasis. These changes result in prolonged action potential duration, abnormal conduction, early and delayed afterdepolarizations, and increased myocardial electrical heterogeneity, all of which heighten the risk of arrhythmias (7).

Additionally, mitochondrial dysfunction can depolarize the mitochondrial membrane and activate sarcolemmal ATP-sensitive potassium (K-ATP) channels. This forms a “current sink” that disrupts the propagation of electrical signals, promoting conduction block and bradyarrhythmias. Oxidative stress, a hallmark of mitochondrial dysfunction, is also implicated in atrial fibrillation (AF) (8). It modifies key calcium channels such as the ryanodine receptor 2 (RyR2), causing calcium leakage in atrial myocytes, which may trigger or exacerbate AF. Animal and human studies have supported the role of mitochondrial ROS in oxidizing RyR2, contributing to intracellular calcium overload and AF pathogenesis.

These pathophysiological processes may present either as isolated cardiac conditions or as part of multisystemic mitochondrial syndromes.

Genetically, mitochondrial dysfunction arises from mutations in mitochondrial DNA (mtDNA) or nuclear DNA (nDNA). However, environmental stressors, aging, and other diseases can also lead to secondary mitochondrial dysfunction, contributing to cardiovascular disease (7). mtDNA mutations manifest in three forms: point mutations in structural or protein-synthesis genes, single or multiple mtDNA deletions as Single Large-Scale Mitochondrial DNA Deletion Syndrome (SLSMDs), and mtDNA depletion (6).

The majority of the mitochondrial inherited lesions are maternally transmitted with incomplete penetrance so that males are disproportionately affected. Point mutations in mtDNA are usually inherited (approximately 75%), though they may occasionally occur *de novo*. These often exhibit low heteroplasmy levels. In contrast, SLSMDs typically arise sporadically during embryonic development and carry a low recurrence risk. Phenotypic expression depends on the degree of heteroplasmy, the proportion of mutant mtDNA relative to normal mtDNA with levels above 70% typically resulting in disease. Single deletions and depletion syndromes are primarily due to mutations in nuclear genes and are often inherited in an autosomal recessive manner (3).

Mitochondrial cardiomyopathies (MCMPs) can result from defects in either genome. The mitochondrial proteome consists of 13 OXPHOS-related proteins, 22 tRNAs, and 2 rRNAs encoded by mtDNA, while over 1,500 proteins are encoded by nuclear DNA. These proteins are crucial for OXPHOS complex assembly, mtDNA maintenance, and overall mitochondrial function. Mutations affecting any of these components can impair OXPHOS, reducing ATP synthesis and disrupting the respiratory chain.

Recent studies have also implicated defects in mitochondrial dynamics, such as fission and fusion processes regulated by non-OXPHOS-related nuclear genes (6), as contributors to MCMPs. These insights continue to expand our understanding of the genetic and molecular basis of cardiac involvement in mitochondrial diseases.

## 3 Cardiac involvement in mitochondrial diseases

Cardiomyopathies (CMPs) are the most frequent cardiac manifestations in MD (Table 1). These myocardial disorders typically occur without other primary cardiac causes, such as coronary artery disease or congenital heart defects. MCMPs are increasingly recognized as secondary forms of CMP, especially in pediatric classifications (9).

**Table 1:** Mitochondrial diseases associated with cardiomyopathies.

Mitochondrial disease subtypes	Gene - variants with cardiac presentation	Age onset	Cardiac disease	Extra-cardiac features	OXPHOS Defects
OXPHOS complex					
Complex I	Subunits AR (*NDUFA2, NDUFA10, NDUFA11, NDUFS2, NDUFS4, NDUFV2)* mtDNA *(MT-ND1, MT-ND2 MT-ND5, G13513A)* *X- NDUFB11* Assembly factors AR *(NDUFAF1, NDUFAF4, ACAD9, FOXRED1, TMEM126B)*	Neonatal Infantile Rarely childhood	HCM Rarely DCM HICM LVNC WPW SCD	Leigh syndrome Leucoencephalopathy Lactic acidosis Epilpesy SNHL Hypoglycemia Ataxia Weakness/EI Micro/macrocephaly Linear skin defects Eyes abnomalities MELAS syndrome LHON	CI
Complex II	Subunits AR *(SDHA, SDHD)* Assembly factors AR *(SDHAF1)*	Prenatal Neonatal Infantile	HCM rarely DCM with LVNC conduction defects	Leigh disease Leukoencephalopathy Cerebellar atrophy Hypotonia/spastic Tetraparesis Ataxia Seizures Carcinoid tumors Cowden-like syndrome Praraganglioma Pheocytochromocytoma	CII
Complex III	Subunits AR (*UQCRFS1)* mtDNA *(MT-CYB)* Assembly factors AR *(BCS1l)*	Infantile	HCM DCM HIMC	Hypotonia Epilepsy Intellectual disability Lactic acidosis Hypoglycemia Growth failure Myopaty/EI Alopecia totalis GRACILE syndrome LHON Septooptic dysplasia	CIII isolated CI+CIII
Complex IV	Subunits AR *(COX6B1);* mtDNA *(MT-CO1, MTCO2, MT-CO3);* X-linked *(COX7B)* Assembly factors AR [*COA5 (=C2orf64), COA6, COX14 (=C12orf62), COX10, SURF1]*	Neonatal Infantile	HCM DCM CHF	Leigh syndrome Lactic acidosis Hypotonia Epilepsy Liver disease Muscle weakness Ataxia Optic atrophy/pigmentary retinopathy SNHL Renal hypoplasia Adrenal hyperplasia Linear skin lesions Microphtalmia LHON	CIV
Complex V	Subunits: AR *(ATP5E);* mtDNA *(MT-ATP6, MT-ATP8)* Assembly factors: AR *(TMEM70)*		HCM DCM WPW	Leigh syndrome Infantile bilateral striatal necrosis Seizures Lactic acidosis Failure to thrive Hyperammoniemia 3 methylglutaconic Aciduria Reversible valproate-Induced pseudoatrohpy Optic and peripheral neuropathy MLASA NARP Retinitis pigmentosa Periodic paralysis	CV
mtDNA DNA, RNA and protein synthesis					
mtDNA replication	AD and AR *(POLG, TWNK, SCL25A4),* AR *(MGME1)* *SUCLG1*	Infantile Childhood	HCM DCM Arrhythmia	Hepatocerebral depletion syndrome Alpers syndrome MNGIE SANDO and SCAE PEO Muscle weakness/EI/atrophy Lactic acidosis Congenital cataracts	CI, CII, CIV, CV
mtDNA trascription	AR *(ELAC2, LRPPRC)* X-linked *(HSD17B10)*	Neonatal Infantile Childhood	HCM CHF	Leigh syndrome Hypoglicemia Dysmophic features NDD Hypotonia/spastic Tetraparesis Choreoathetosis Seizures FFT Lactic acidosis Optic atrophy SNHL ↑ u-2-methyl-3 Hydroxybutyrate ↑ u tiglylglycine	CI, CII, CIV, CV Abnormal cristae RRF
Mitochondrial translation					
tRNA transfer	AR (*MT-TA, MT-TG, MT- TL1, MT-TP*)	Neonatal Infantile	HCM DCM HICM		CI, CII, CIV, CV
tRNA modificators	AR *(GTPBP3, MTO1, TRMT5, TRMU, GFM1, RMND1*)				CI, CII, CIV, CV
tRNA aminoacylation	AR (*AARS2, GARS, HARS2, IARS2, KARS, LARS2, PARS2, WARS2, VARS2, YARS2*)				CI, CII, CIV, CV
Start t-RNA	AR (*MTFMT)*				
Ribosomal RNA and protein synthesis	mt-DNA *(MT-RNR1, MT-RNR2)* AR *(MRPS22, MRPL3MRPL44)*				CI, CII, CIV, CV
Regulation	AR *(GFM1, RMND1, TSFM)*				CI, CII, CIV, CV
Cofactors					
Coenzyme Q_10_	AR *(COQ2, COQ4, COQ9, PDSS1)*	Infantile	HCM Valvular disease	Infantile encephalomyelopathy Nephropaty SNHL Optic atrophy, peripheral neuropathy, obesity, livedo reticularis	CI+CIII, CII+CIII
Iron-sulphur cluster	AR *(BOLA3, FXN, ISCU, NFU1, LIAS)*	Infantile	HCM DCM Pulmonary hypertension		
Obstructive vasculopathy	Lactic acidosis Seizures Myopathy Optic atrophy Developmental regression Leukodystrophy	CI, CII, CIII			
S-adenosyl methionine	AR *(SLC25A26)*	Infantile		Early death Respirator insufficiency Hydrops Cardiopulmonary failure Slow and progressive muscle weakness	CI, CIII, CIV, CV
Copper	AR *(COA6, COX10, COX15, SCO1, SCO2)*	Neonatal, infantile	HCM LVNC	Leigh syndrome encephalopathy Proximal renal tubulopathy	CIV
Inhibitors					
	AR *(ECHS1)*	Infantile	HCM DCM arrhytmia CHD	NDD Leigh syndrome Hearing impairment ↑ u-3-methylglutaconic acid ↑u-2-methyl-2,3-dihydroxybutyrate	CI, CIII, CIV, CI+CIII
Mitochondrial homeostasis					
	AR (*AGK, DNJC19, C1QBP*) X-linked (TAZ)	Infantile	HCM DCM LVNC Arrhythmias	Myopathy/EI Growth failure Neutropenia ↑ 3-methylglutaconic acid Congenital cataracts Lactic acidosis Kidney failure PEO	CI, CIII, CIV

AD, autosomal dominant; AR, autosomal recessive; CI, complex I; CII, complex II; CIII, complex III; CIV, complex IV; CV, complex V; CHF, congestive heart failure; DCM, dilated cardiomyopathy, EI, exercise intolerance; FFT, failure to trive; HCM, hypertrophic cardiomyopathy, HICM, histiocytoid cardiomyopathy; LHON, Leber hereditary optic neuropathy; LVNC, left ventricular non compaction; MELAS, melas mitochondrial encephalomyopathy lactic acidosis and stroke like episodes; MNGIE, mitochondrial neurogastrointestinal encephalopathy syndrome; MLASA, mitochondrial myopathy, lactic acidosis and sideroblastic anemia; NARP, neuropathy, ataxia, and retinitis pigmentosa; NDD, neurodevelopmental delay OXPHOS, oxidative phosphorylation; PEO, progressive external ophthalmoplegia; SANDO, sensory ataxic neuropathy, dysarthria, and ophthalmoparesis; SCAE, spinocerebellar ataxia with epilepsy; SCD, sudden cardiac death; SNHL, sensorineural hearing loss; RRF, ragged red fibers; U, urinary; WPW, Wolff–Parkinson–White.

The main types of MCMPs include hypertrophic (HCM), dilated (DCM), and left ventricular non-compaction (LVNC) cardiomyopathies. Less commonly, restrictive cardiomyopathy (RCM) and histiocytoid cardiomyopathy (HICMP) are observed (10) (Table 2). The clinical presentation varies widely from severe neonatal onset to asymptomatic childhood cases and can be the first or sole manifestation of MD, although they more commonly occur alongside multi-organ involvement (11).

**Table 2:** Classical syndromic presentations of MD according to age of onset.

Disease	Features	Onset
Congenital Lactic Acidosis	Fatal in infantile lactic acidosis in children with complex I deficiency	Infantile
Infantile-onset mitochondrial DNA depletion syndromes (MDDS)	Defined by a quantitative reduction in the absolute number of mtDNA copies. Infants with severe MDDS typically have <10% residual mtDNA in the affected tissues. *Clinical presentation* includes: myopathic, encephalomyopathic, hepatocerebral or multisystem disorder or underlying genetic mechanism (defect of the mtDNA replication apparatus, mtDNA repair, nucleoside metabolism or mitochondrial dynamics)	Infantile
Benign reversible mitochondrial myopathy	Characterized by lactic acidosis and a profound myopathy affecting the limbs and respiratory muscles, often leading to gastrostomy feeding and artificial ventilation. There is usually a remarkable recovery with supportive care, with only mild residual myopathy persisting into adulthood	Infantile (6 weeks-3 months)
Pearson syndrome	Caused by a single large scale mtDNA deletion Characterized by transfusion-dependent anaemia and lactic acidosis, variously associated with feeding difficulties, and develop mental retardation. The need for transfusions gradually becomes less frequent over time with complete resolution of the anaemia typically around two years of age. Other bone marrow lineages are frequently affected leading to neutropaenia, thrombocytopenia or pancytopenia (11). Some patients present with severe growth restriction, metabolic acidosis and liver failure with an extremely high mortality. In patients who survive, resolution of the anaemia is followed by a period of relatively good health before the onset of multisystem problems in the spectrum of Kearns-Sayre syndrome	Early childhood
Barth syndrome	X-linked disorder caused bay mutations in TAZ, encoding tafazzin. Characterised by 3-methylglutaconic aciduria associated with DCM, skeletal myopathy (mainly proximal), impaired growth and (cyclic) neutropenia. Other cardiac features are endocardial fibroelastosis, left ventricular non-compaction and HCM	Infantile
Sengers syndrome	Autosomal recessive disorder caused by mutations in AGK encoding acylglycerol kinase, Clinical presentation: congenital cataracts, HCM, skeletal myopathy, exercise intolerance and lactic acidosis.	Childhood
Leigh syndrome	Caused by mutations in at least 75 different genes. Typical findings include symmetrical spongiform degeneration of the corpus striatum and brainstem with demyelination and vascular proliferation. Clinical presentation: psychomotor retardation or regression, hypotonia, respiratory abnormalities, seizures, and a general inability to thrive. Other features include encephalopathy, epilepsy, movement disorders, risk of aspiration with feedings due to dysphagia and bulbar weakness, nystagmus, apnoea and ataxia. Less frequent symptoms are diabetes, cardiomyopathy, anaemia, renal failure, vomiting and diarrhoea.	Infantile Childhood
Alpers-Huttenlocher syndrome	Characterized by focal motor seizures that evolve into bilateral seizures and often into continuous partialis epilepsy and status epilepticus. The prognosis is extremely poor; most cases have a rapidly progressive course leading to death from status epilepticus or liver failure within a few months of presentation	Infancy Early childhood
Kearns-Sayre syndrome	Progressive external ophthalmoplegia, pigmentary retinopathy and heart block. Other symptoms comprise sensorineural hearing loss, renal tubulopathy, gastrointestinal dysmotility, endocrine disorders, cardiomyopathy, basal ganglia calcification and leukoencephalopathy, often associated with folate deficiency in the brain for reasons that remain unclear.	Before 20 years of age
Mitochondrial myopathy, Encephalopathy, Lactic Acidosis and Stroke-like episodes (MELAS)	Characteristics are lactic acidosis and stroke-like episodes, usually heralded by migraine, homonymous hemianopia or quadrantanopia and convulsions. Metabolic strokes differ from arterial ischemic strokes because they do not follow a vascular territory. Additional symptoms include short stature, cognitive decline, exercise intolerance, sensorineural hearing loss, ptosis, optic atrophy, gastrointestinal dysmotility and diabetes mellitus.	Two peaks: one as infantile form followed by another peak around 35 years
Myoclonic epilepsy with raged red fibres (MERRF)	caused by a single mtDNA mutation in the MT-TK gene in most cases, Presents in childhood with ataxia, sensorineural hearing loss and endocrine disorders. Myoclonus may be a late presentation. Other clinical features of MERRF include cognitive impairment, multiple lipomatosis, ptosis/PEO, myopathy, peripheral neuropathy and cardiomyopathy.	Often in childhood
Juvenile-onset POLG syndromes	Mutation in POLG gene with overlapping phenotypes. Two acronyms have been used to describe the main POLG disease. Phenotypes occurring in adolescence: myoclonic epilepsy, myopathy, sensory ataxia (MEMSA) and ataxic neuropathy syndrome (ANS)	Ranges from infancy to late adulthood
Leber's hereditary optic neuropathy (LHON)	Characterized by painless subacute central visual loss affecting both eyes sequentially. Most patients have isolated ophthalmological symptoms, but dystonia or cardiac conduction problems such as Wolff-Parkin-son-White syndrome may be associated	Second or third decade of life
Progressive external ophthalmoplegia (PEO)	The current consensus is that there is a continuous spectrum of clinical phenotype associated with SLSMDs, ranging from Pearson syndrome at the most severe end through Kearns–Sayre syndrome to isolated PEO as the mildest manifestation of SLSMDs	Isolated finding is a rare presentation in childhood
Neuropathy, ataxia, and retinitis pigmentosa (NARP)	Most patients experience numbness, tingling, or pain in the arms and legs (sensory neuropathy); muscle weakness; and problems with balance and coordination (ataxia). Many affected individuals also have vision loss caused by deterioration in the retina, a condition called retinitis pigmentosa. Signs and symptoms usually worsen over time	Typically begins in childhood or early adulthood

mtDNA, mitochondrial DNA; FILA, Fatal in infantile lactic acidosis; MDDS, mitochondrial DNA depletion syndromes; SLSMD, single large scale mtDNA deletion; KSS, Kearns-Sayre syndrome; TAZ, tafazzin; DCM, dilated cardiomyopathy; HCM, hypertrophic cardiomyopathy; MELAS Mitochondrial myopathy, Encephalopathy, Lactic Acidosis and Stroke-like episodes; MERRF, Myoclonic epilepsy with raged red fibres; MEMSA myoclonic epilepsy, myopathy, sensory ataxia syndrome; ANS, ataxic neuropathy syndrome.

Symptoms range from mild to severe and include heart failure (HF), arrhythmias, and sudden cardiac death (SCD). Diastolic dysfunction and HF with preserved ejection fraction may appear early in the disease course (3, 12–14). Metabolic decompensation triggered by stressors like infection or surgery can precipitate or exacerbate cardiac symptoms, including acute HF (15). Children with both MD and CMPs have significantly higher mortality compared to those without cardiac involvement (12, 15, 16).

MCMPs can be isolated or occur without a prior diagnosis of MD, making them a potential first clue to an underlying mitochondrial disorder (16). Some ae- and sex-related trends have been observed: neonatal presentations typically involve HCM, LVNC is more common in males, and in some females, it may emerge during pregnancy and resolve later. RCM remains a rare phenotype in MD (10, 11).

Beyond CMPs, patients may experience arrhythmias, conduction system defects (in 5%–10% of MD patients), pulmonary hypertension, pericardial effusion, aortic root dilation, and coronary artery disease. Conduction defects are often linked to neuromuscular forms of MD, particularly those associated with single large-scale mtDNA deletions (SLSMDs) (3).

Biomarker such as brain natriuretic peptides is frequently elevated in MD and correlate with cardiac involvement. MD significantly increases the risk of adverse cardiovascular outcomes, including heart failure, in-hospital death, and overall morbidity (17).

While MDs account for a minority of CMP cases, identifying a metabolic etiology has critical implications for prognosis, treatment, and genetic counselling (18–20). Major MD subtypes linked to CMPs include lysosomal and glycogen storage diseases, organic acidurias, fatty acid oxidation defects, and OXPHOS disorders (8, 12, 13).

## 4 Cardiac phenotype

### 4.1 Hypertrophic cardiomyopathy

HCM is a rare pediatric condition, most often idiopathic or caused by sarcomeric gene mutations (about 60%) (9–21). However, over a third are phenocopies, commonly related to MD. HCM is the most frequent form of mitochondrial cardiomyopathy (MCMP), seen in around 40% of cases. Mitochondrial HCM often presents with concentric hypertrophy, rarely involves left ventricular (LV) outflow tract obstruction, and more frequently progresses to LV systolic dysfunction (9, 12, 21).

The Pediatric Cardiomyopathy Registry reports worse outcomes in MD-related HCM: a 5-year survival of 42%, compared to 94% in idiopathic cases diagnosed after one year of age. MD HCM is typically diagnosed earlier, with more frequent heart failure at onset, increased end-diastolic dimension z-scores (*p* = 0.012), reduced fractional shortening, and a more concentric hypertrophy pattern (*p* < 0.001). Mortality is highest in those diagnosed within the first year of life (22).

HCM is seen in syndromic MDs such as Mitochondrial Encephalomyopathy, Lactic Acidosis, and Stroke-like episodes (MELAS), Myoclonic Epilepsy with Ragged Red Fibers (MERRF), **Chronic Progressive External Ophthalmoplegia** (CPEO), Leigh syndrome, and Neuropathy, Ataxia, and Retinitis Pigmentosa (NARP), as well as in respiratory chain complex deficiencies, CoQ10 deficiency, mitochondrial depletion syndromes (e.g., MNGIE), and β-oxidation disorders. Isolated HCM is associated with mutations in nuclear-encoded subunits (NDFUF2, NDUFV2) and assembly cofactors (ACAD9, occasionally NDUFAF1). Complex I deficiencies can also manifest as dilated cardiomyopathy (DCM), histiocytoid cardiomyopathy (HICMP), LV non-compaction (LVNC), and conduction abnormalities like Wolff-Parkinson-White (WPW) syndrome (23).

Leigh Syndrome, a neurodegenerative condition, presents before age 2 with hypotonia, seizures, respiratory issues, neurodevelopmental delay, ataxia, and lactic acidosis. Later-onset forms may include behavioral issues, psychiatric symptoms, or cognitive decline. Around 20% of patients develop heart disease, including HCM or DCM in about 10%, more often in mtDNA-related cases. Cardiac involvement may emerge at any stage (5, 24).

MERRF is a maternally inherited disorder marked by epilepsy, lactic acidosis, muscle weakness, ataxia, deafness, and dementia. Additional symptoms include short stature, optic atrophy, neuropathy, CMP, and renal issues (25). Elevated lactate/pyruvate levels are common. Muscle biopsies reveal ragged red fibers. Cardiac manifestations include HCM and arrhythmias: AV block, WPW, supraventricular tachycardia (SVT), and RBBB. The m.8344A>G mutation in MT-TK accounts for over 80% of cases. Other mutations include MT-TF, MT-TH, MT-TI, MT-TL1, MT-TP, MT-TS1, and MT-TS2 (26, 27).

MELAS syndrome often includes HCM, which worsens prognosis, especially in childhood-onset cases (12, 28). Though rare in infancy, neonatal presentations are reported. Severity correlates with mutation load (9, 21). Like MERRF, MELAS is associated with conduction defects and ventricular pre-excitation.

MIDD (Maternally Inherited Diabetes and Deafness) is defined by diabetes and sensorineural hearing loss (SNHL). Other features may include brain atrophy, ptosis, GI dysmotility, and pseudo-obstruction. Cardiac involvement includes HCM in 15%–30%, LV hypertrophy in 55%, and conduction issues (WPW, sinus node dysfunction, atrial fibrillation) in 27%. The m.3243A>G mutation in MT-TL1 is found in 80% of cases, with less frequent variants in MT-TE and MT-TK (29).

MNGIE (Mitochondrial Neurogastrointestinal Encephalopathy) presents in adolescence to early adulthood with GI dysmotility, leukoencephalopathy, ophthalmoplegia, neuropathy, and failure to thrive. Cardiac manifestations include ECG abnormalities, LV hypertrophy, prolonged QT, SVT, and sudden cardiac death (SCD). It results from TYMP mutations or, less commonly, POLG, leading to mitochondrial depletion syndrome (29).

Sengers Syndrome, linked to AGK gene variants, involves congenital cataracts, skeletal myopathy, exercise intolerance, HCM, and urinary 3-methylglutaconic aciduria. AGK plays a role in mitochondrial lipid homeostasis and protein import (29).

TMEM70 deficiency, the most common nuclear ATP synthase defect, causes neonatal lactic acidosis, respiratory distress, hypotonia, HCM, developmental delay, microcephaly, and persistent pulmonary hypertension. WPW syndrome may also be present. HCM has high penetrance, reported in 89% to 100% of cases in different series (29, 30).

Friedreich's Ataxia (FRDA) is an autosomal recessive condition caused by GAA repeat expansions in the FXN gene, affecting mitochondrial iron metabolism. It's the most common inherited ataxia (incidence 1:30,000; prevalence 1:50,000). Symptoms include ataxia, incoordination, CMP, diabetes, and scoliosis, typically starting in mid-childhood. Larger GAA expansions correlate with earlier onset, severe disease, and more prominent cardiac involvement (31–33).

HCM develops in two-thirds of FRDA patients, often after neurological signs. It progresses from concentric remodeling to hypertrophy, and eventually LV dilation and dysfunction, leading to HF or cardiac death. HCM is typically concentric and non-obstructive with LV wall thickness under 15 mm. Echocardiography may show a granular texture, and early reduced global longitudinal strain (34–36). Fibrosis is reported early in the disease (23).

Cardiac fibrosis increases the risk of conduction blocks and arrhythmias. Atrial arrhythmias like flutter or fibrillation are seen, though less frequently than ventricular arrhythmias. Some patients benefit from pacemakers or ICDs (37). HCM may evolve into DCM, which is associated with a poor prognosis. LV systolic function is typically preserved until late-stage disease. While many FRDA patients are asymptomatic, palpitations or dyspnea are common presenting complaints. Cardiac dysfunction is the leading cause of death in FRDA (34).

A review of 61 FRDA patients reported that 59% of deaths were cardiac-related: 30% from HF and 20% from arrhythmias. Most deaths occur in the third or fourth decade, often earlier for cardiac deaths (38). A UK cohort of 78 pediatric FRDA-HCM patients showed 96.5% 5-year survival and 80.8% at 10 years. HCM was diagnosed in all, with 40% diagnosed before age 10, and a few even before the neurological diagnosis of FRDA. Cardiac-related deaths occurred mostly in those with shorter disease duration (<10 years), while those living longer than 20 years had a lower risk (39, 40).

### 4.2 Dilated cardiomyopathy

DCM is less frequent than HCM in MD and can occur as a progression from pre-existing HCM, with a dilated and hypokinetic LV. It's associated with CPT II deficiency (10), Kearns-Sayre syndrome, MELAS, MERRF, complex I and cytochrome C oxidase deficiencies, Leigh syndrome, MIDD, and LHON (41).

DCM is also seen in organic acidurias MDs of organic acid metabolism. Propionic aciduria shows DCM in up to 40%, often during adolescence with hypokinetic, dilated LV and frequent long QT syndrome (up to 70%), increasing risk for malignant arrhythmias and rare SCD. Cardiovascular (CV) involvement worsens outcomes, though liver transplant may reverse cardiomyopathy (15). Methylmalonic aciduria is rarely linked to cardiomyopathy, but both HCM and DCM phenotypes have been reported (10).

Inborn errors of metabolism (IEMs) account for 4% of pediatric DCM cases in the Pediatric Cardiomyopathy Registry (42). Among these, mitochondrial disorders represent 46% and Barth syndrome 24%. Most were diagnosed in infancy (52%). Despite severity, IEM-related DCM had the best 5-year composite outcome (78% free from death or transplant), with 94% free from transplant, compared to 62% in idiopathic/familial cases (43). This favorable outcome is partly due to the fact that transplantation is often not pursued in these patients.

### 4.3 Left ventricle non-compaction

LVNC is a recognized cardiac manifestation in MD, especially in children, often as part of multisystem involvement. It has been associated with the m.3398T>C MTND1 variant, making LVNC a possible phenotype in MELAS syndrome (21).

Barth syndrome is the primary MD linked to DCM-LVNC in male children. It is an X-linked multisystemic disorder caused by TAZ gene mutations, which encode tafazzin a phospholipid transacylase involved in cardiolipin remodeling. This results in decreased and structurally abnormal cardiolipin levels. Barth syndrome presents variably with dilated cardiomyopathy (DCM), LVNC, skeletal myopathy, growth delay, neutropenia, and elevated urinary 3-methylglutaconic acid. Not all features are present in every patient, and phenotype may change with age. DCM is present in over 70% of affected males, often before age 5, with some fetal presentations reported (44–46).

LVNC features, particularly prominent trabeculations, are seen in about half of Barth syndrome cases. However, other cardiac forms such as hypertrophic cardiomyopathy (HCM) or endocardial fibroelastosis may also occur (46, 47). Disease onset may mimic viral myocarditis or DCM, particularly if triggered by infection. Barth syndrome should be considered in male patients with DCM and neutropenia, especially when attributed to viral illness. During mid-childhood, cardiomyopathy may stabilize, and general health improves. Early response to standard medical therapy is often positive, though progression may necessitate transplantation (47, 48). Complications can include heart failure, thromboembolism, and arrhythmias (45, 49, 50). Prognosis is significantly influenced by the severity of cardiomyopathy. Patients are at high risk for conduction defects, supraventricular and ventricular arrhythmias, which may lead to sudden cardiac death (SCD). These rhythm disturbances may not correlate with the degree of cardiac dysfunction and can present at any age (47, 51). Another MD associated with LVNC is **dilated cardiomyopathy with ataxia (DCMA) syndrome**, caused by DNAJC19 mutations. It features DCM or LVNC, non-progressive cerebellar ataxia, testicular dysgenesis, and growth failure (10).

**Combined methylmalonic aciduria and homocystinuria** due to MMACHC mutations is also linked to LVNC. Other features include failure to thrive, encephalopathy, hydrocephalus, acidosis, renal dysfunction, and thrombosis. Fetal presentations with DCM and LVNC have been reported (10, 52, 53). Mutations in **C1QBP**, which encodes a mitochondrial protein involved in respiratory chain function, cause severe neonatal or later-onset DCM with respiratory chain deficiencies. In infancy, this may lead to heart failure, kidney failure, lactic acidosis, and early death. In adults, C1QBP mutations can manifest as cardiomyopathy, LV hypertrophy, progressive external ophthalmoplegia (PEO), and myopathy (52, 53).

### 4.4 Restrictive cardiomyopathy

RCM is a rare form in MD. It has been reported in association with MIDD due to the m.3243A.G mutation, and as the only clinical finding in a subject with the m.1555A.G mutation (multiple respiratory chain complex deficiency—MT-RNR1 enc. mt-12S rRNA) (10, 21, 54).

### 4.5 Cardiac conduction defects

Pearson syndrome (PS) presents in infancy with bone marrow failure, pancreatic insufficiency, and endocrine dysfunction, often resulting in early mortality. Survivors may develop **Kearns-Sayre syndrome (KSS)**, where cardiac involvement becomes prominent, primarily due to progressive conduction defects. These can range from isolated atrioventricular block (AVB), right and left bundle branch blocks (RBBB, LBBB), to complete AVB requiring pacemaker implantation. KSS is a multisystem neurodegenerative MD characterized by progressive external ophthalmoplegia (PEO), pigmentary retinopathy, and onset before age 20. Other features include ptosis, growth failure, leukoencephalopathy, myopathy, neuropathy, and endocrine abnormalities (55). Cardiac involvement occurs in ∼50% of patients and is the main determinant of prognosis. There is no clear link between age of onset and cardiac symptoms (55).

**Conduction abnormalities**, including fascicular and bundle branch blocks, often progress unpredictably to complete AVB, frequently the cause of sudden death. Syncope is a common first symptom, occurring in about 50% of patients, while sudden cardiac death (SCD) is reported in up to 20% (55–57). Because risk stratification remains unclear, routine cardiac monitoring, including 12-lead ECGs and Holter monitoring, is essential for early detection and intervention. Ventricular arrhythmias such as long QT, torsades de pointes, and both supraventricular and ventricular tachycardias have been reported. In some cases, management requires an implantable cardioverter-defibrillator (ICD). ESC guidelines recommend (class IIaC) pacemaker implantation in patients with KSS who show PR prolongation, any AVB, or intraventricular conduction delays (58). Cardiac anomalies may present as the first symptom in pediatric KSS cases. Di Mambro et al. found that complete or advanced AVB occurred in 40% of pediatric KSS patients, with left anterior fascicular block often preceding RBBB in the progression to complete AVB (59). Notably, no significant difference in conduction anomalies was found between classical and non-classical mtDNA deletions. Given that syncope or SCD can be initial manifestations, early and proactive management, including prophylactic pacemaker implantation, is advised to prevent fatal events. Electrophysiological studies show primary abnormalities in the AV node-His-Purkinje system, including shortened atrial-His conduction and prolonged H-V intervals (60). Histopathologic findings have revealed fatty infiltration and fibrosis in the bundle branches and nodes (61). Progressive mtDNA depletion in cardiomyocytes is suspected to underlie the conduction system deterioration (62–64). Because of the progressive nature of KSS, complete AVB or cardiac arrest may occur without warning (65), further emphasizing the need for early and routine cardiac surveillance.

**CPEO**, a milder phenotype of the SLSMD spectrum, presents after age 20 with ptosis and ophthalmoplegia, and may also include conduction defects similar to those seen in KSS (23).

## 5 When to suspect MD

**Figure 1:**
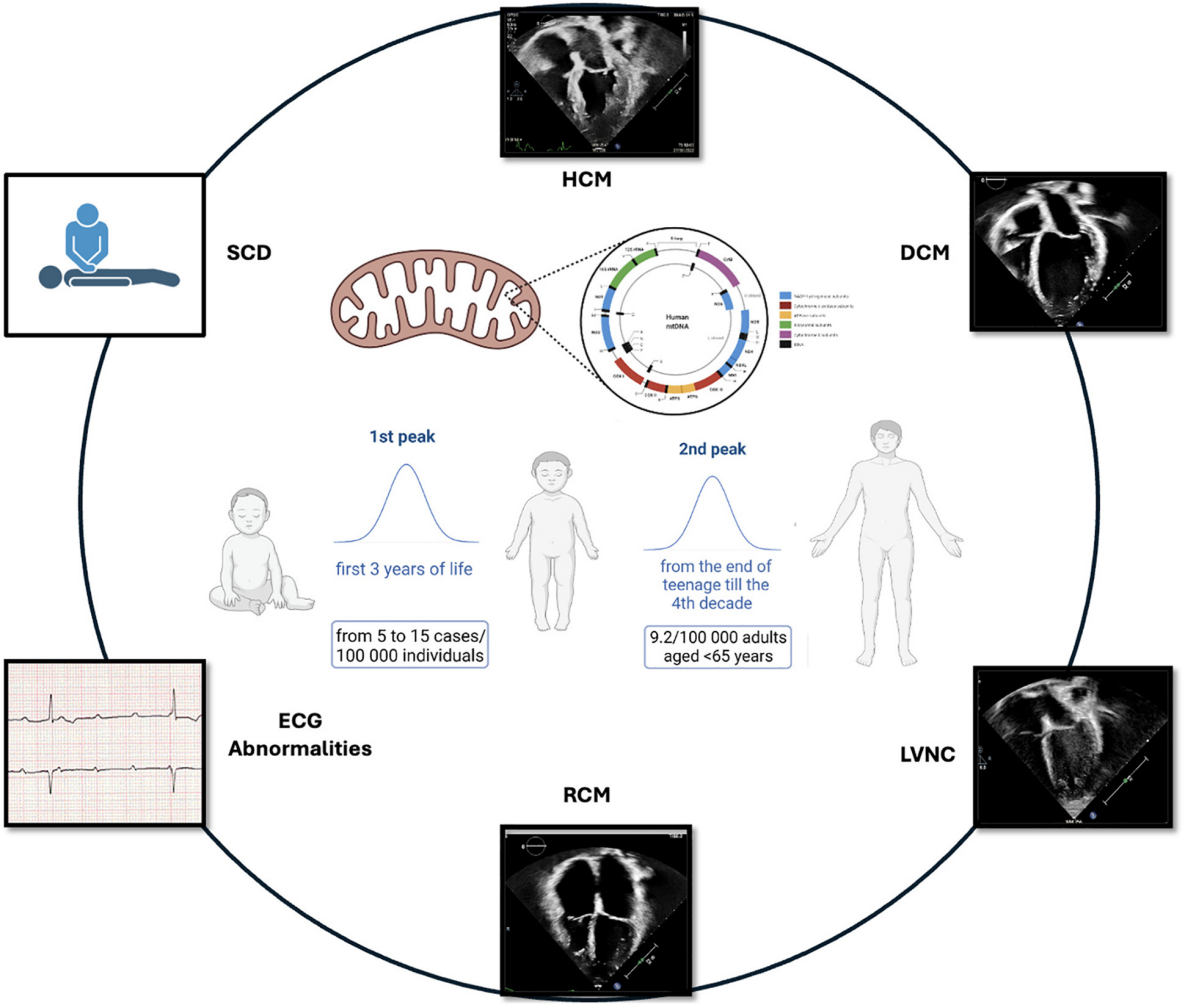
Bimodal distribution of MCMPs onset.

MD should be suspected when neuromuscular and non-neuromuscular symptoms coexist, especially with rapid progression or multi-organ involvement. Red flags include insulin-dependent diabetes, sensorineural deafness, growth failure, muscle weakness, and kidney disease not explained by diabetes (3). Neurologic symptoms are common, though up to 40% of patients lack cognitive decline (3, 4). MD are clinically heterogeneous and may manifest at any age. A bimodal distribution of onset is observed, with the first peak occurring in early childhood and a second, adult-onset phase beginning in late adolescence and extending through the fourth decade (66, 67) (Figure 1). **MELAS syndrome** is the prototype of systemic MD and represents 25% of cases. MCMPs occur in one-third of MELAS patients (1, 2).

### 5.1 MD in children

Children often exhibit a more acute onset than the progressive course seen in adults (67). Neonatal-onset forms often have severe courses, with up to 50% annual mortality (68). Classical pediatric MD presentations by age are outlined in Table 3 @ (38, 39). Cardiac involvement significantly worsens prognosis (69). A retrospective study showed that children with CMP had far worse survival (18%) than those with neuromuscular symptoms alone (95%) (70). At Texas Children's Hospital, 40% of 113 pediatric MD patients had cardiac disease: 58% had HCM, 29% DCM, and 13% LVNC. CMP drastically reduced survival: 18% at 16 years vs. 95% in those without CMP (*p* < 0.0001) (11). An Italian study of 85 pediatric MD patients found mortality was 45.1% in those with CV involvement vs. 21.8% without (*p* < 0.01) (69).

**Table 3:** Organ-specific involvement in mitochondrial disease.

Disease-related organ involvement	Clinical presentation
Skeletal, Muscle	Frequent symptom in childhood is exercise intolerance, characterised by muscle cramps and fatigue on exertion, but patients may also present with vomiting after exercise related to lactic acidosis.
Encephalomyopathies	Epilepsy has a prevalence of around 40%–60% in paediatric MD and is associated with defects in more than 140 mitochondrial disease genes, representing an adverse prognostic factor.
Dementia	A red flag sign of childhood neurodegenerative disease is regression or loss of previously achieved milestones. When regression is triggered by illness, surgery, stroke or epilepsy, mitochondrial disease—especially POLG or Leigh syndrome— may be the cause. Neuropsychological testing may help in determining the degree of cognitive impairment. Children may have frank behavioural changes, or the cognitive decline may be similar to that seen in Alzheimer or Parkinson disease
Neuropathies	Mitochondrial neuropathy can be axonal motor, axonal sensorimotor, sensory ataxia or demyelinating
Eye diseases	In addition to eye muscle involvement (ptosis/PEO), any layer of the eye can be affected, from corneal opacification (Pearson/KSS) to cataracts (Sengers syndrome), optic neuropathy (LHON) and pigmentary retinopathy (NARP).
Hearing loss	Sensorineural and usually bilateral, symmetrical and progressive. It may be of cochlear origin or caused by auditory neuropathy. Around 40% of patients with MD present hearing loss.
Endocrine disorders	Any endocrine organ can be affected by MD, because of decreased intracellular production or extracellular secretion of hormones. Diabetes mellitus is the most frequent endocrine manifestation of MD in adults, but in childhood it is most often seen together with growth hormone deficiency, adrenal insufficiency, hypothyroidism and hypoparathyroidism.
Renal disease	In children renal involvement present most commonly in the form of proximal tubulopathy (mutations in RRM2B) or as steroid-resistant nephrotic syndrome while in adulthood focal segmental glomerulosclerosis is more common. The tubulopathy may be of the Fanconi type or more similar to Gitelman's syndrome with severe hypomagnesemia.
Gastrointestinal disease	Gastrointestinal symptoms are a frequent feature of MD in childhood and may be among the first clinical features, presenting with vomiting, feeding difficulties and growth retardation.
Hepatopathy	Liver involvement is a frequent feature of early-onset MD. Presentation may be with acute or chronic liver failure and is typically progressive and fatal. Histological features of mitochondrial liver disease include micro- and macrovesicular steatohepatitis, which may progress to micro- or macronodular cirrhosis.
Haematological involvement	Pearson syndrome is the most common cause of mitochondrial sideroblastic anaemia. Anaemia is a frequent finding in children with other MD and has an adverse prognostic impact.
Immune dysfunction	B-cell immunodeficiency appears to be a consistent feature of TRNT1 deficiency, which is sometimes known by the acronym SIFD, for sideroblastic anaemia, immunodeficiency, fever and developmental delay. This condition is characterised by recurrent debilitating episodes of severe fever.
Dysmorphology	Characteristic facial appearances have been reported for some MDs but the rarity of these diseases makes it impossible to identify a characteristic facies such that MD can be recognised by facial appearance alone.
Skin and hair abnormalities	Hypertrichosis is often a prominent feature in children with SURF1 deficiency.^38^ Excess hair is typically observed on forearms, thighs, shins and upper back. Hair abnormalities (pili torti) have been reported in Bjornstad syndrome, caused by deficiency of the complex III assembly factor BCS1l.^39^ Interestingly, alopecia *totalis* has recently been reported in children with deficiency of the complex III subunit UQCRFS1.

### 5.2 MD in adulthood

Population studies estimate that 9.2 per 100,000 adults (<65 years) are clinically affected by MD, with another 16.5 per 100,000 genetically predisposed (71). mtDNA mutations are responsible for most adult-onset MD, while nDNA mutations account for about one-third of cases. Diagnosing MD in adulthood is particularly challenging due to oligosymptomatic presentations, such as isolated diabetes or migraine, or complex multisystem involvement (neurological, cardiac, endocrine, etc.). The weak genotype-phenotype correlation, rarity of the disease, and slow progression frequently cause diagnostic delays, often decades after symptom onset.

Cardiac involvement is a critical prognostic factor in MD, strongly predicting morbidity and mortality (11, 72–74). In adults, heart disease may present as CMPs or conduction abnormalities (“electropathy”).

HCM is the most common phenotype, seen in up to 40% of adult MD patients, and may mimic sarcomeric HCM (72, 75, 76). DCM can occur but usually reflects disease progression from pre-existing HCM (77, 78). RCM is rare but has been linked to specific mtDNA mutations such as m.3243A>G (MT-TL1) and m.1555A>G (54, 79).

Electropathy, including conduction system disease and ventricular pre-excitation, is another hallmark of adult MD, particularly in mtDNA-related disorders. Conduction defects increase with age and can progress to high-grade AV block. WPW syndrome is reported in mtDNA diseases, though less frequently than in other inherited conditions like PRKAG2-related disease (21).

The natural history of cardiac involvement in adult MD is difficult to define due to the broad phenotypic variability. In a large cohort of 260 adult MD patients (median age 43), 30% had cardiac disease, and 10% experienced major cardiovascular events over a median 7-year follow-up (80). Predictors included intraventricular conduction defects, LV hypertrophy, premature ventricular complexes, and diabetes. Two of the most common adult-onset MD types include the m.3243A>G mutation (MT-TL1 gene) and single large-scale mtDNA deletions, which underlie syndromes like MELAS and Kearns-Sayre. MELAS often presents in childhood, but CMP becomes prominent in adulthood (81, 82). In patients with the m.3243A>G mutation, LV hypertrophy has been reported in 38%–56% of adults (76). A longitudinal study showed that the degree of hypertrophy positively correlated with LV dilation and negatively with systolic function (83). Cardiac conduction abnormalities, including WPW, have been reported in 13%–27% of MELAS patients (83, 84, 85). While symptomatic WPW warrants electrophysiologic evaluation and potential ablation, asymptomatic patients may benefit from risk stratification, especially if there's a family history of SCD (86).

Serious cardiac complications in MELAS include malignant arrhythmias, heart failure, and SCD, which may even occur in the absence of overt CMP (87, 88). These findings emphasize the need for regular cardiac surveillance, particularly in carriers of the m.3243A>G mutation. **Leber hereditary optic neuropathy (LHON)** is another common adult-onset MD, primarily affecting retinal ganglion cells, leading to central vision loss (89, 90). In LHON, HCM and ECG abnormalities have been documented, although cases of DCM and LVNC have also been reported (90). However, most data come from case reports or small series, limiting broader understanding.

## 6 Diagnosis of MD in case of cardiac disease

The diagnosis of MD is complex and requires an integrated diagnostic approach based on accurate family history, clinical and biochemical screening a more specific histopatological studies and molecular genetic testing (91). Due to the broad clinical and progressive presentation, an accurate and multisystemic evaluation with screening of all the organs and systems involved is therefore recommended (Table 4). Cardiovascular presentation may be at any age and the analysis of other organ, including the brain, eye, skeletal muscle, and heart, is essential to suspect MD. On the other way, the presence of signs and symptoms of CMPs associated to a multisystemic involvement should raise the suspicion of MD and the need to refer the patients to physician and genetics with expertise in MDs.

**Table 4:** First and second level laboratory examinations in the suspicion of MCMPs.

First level	Second level
CBC	Organic acid (u)
AST, ALT, bilirubin, GGT	Amino-acids (pl)
CPK	Acylcarnitines (DBS, pl)
Iron, Ferritin, Transferrin	Lipid panel and Cardiolipin
ALP	FGF21, GDF15
Lactate, pH	
Ammonia	
Coagulation factors	

CBC, complete blood count; FGF21, fibroblast growth factor 21; GDF15, growth differentiation factor 1.

An integrated diagnostic approach should be followed to address a correct and early diagnosis of MCMPs.

The first step is represented by a careful physical examination to assess cardiac function and all the other organs (i.e., deafness, myopathy, peripheral neuropathy, eyes disease, ptosis, ataxia) potentially be associated to CMPs. Extensive pedigree analysis should be carry-out to identify possible maternal transmission suggestive for mtDNA alterations.

The first level laboratory investigations with complete blood cell count, hepatic, nephro-tubular and endocrinological function including basal and after-stimulation adreno-cortical function.

The second level investigations consist of metabolic profile including lactate, plasma amino-acids, urinary organic acids, plasma and dried blood spot (DBS) acylcarnitine, lipid panels and analysis of cardiolipin and cardiolipin species. Elevated lactate is highly suggestive of MD, but a normal lactate doesn't rule out a diagnosis of MD. Hypoglycaemia can be associated with suspected MD especially in the presence of metabolic acidosis with elevated lactate, high urinary excretion of lactate, Krebs intermediates and keton bodies. At plasma amino-acids elevation of alanine and proline reflecting elevated lactate level and at urinary organic acid, the presence of 3-methylglutaconic acid are highly suggestive of MD. Elevated levels of two new biomarkers FGF-21 and GDF-15 have been also recently identified as markers of MDs (Table 5).

**Table 5:** Cardiac phenotype correlated to MD.

Cardiac phenotype	Mitochondrial disease
HCM	ACAD9, CPEO, Leigh syndrome, LHON, FRDA, MELAS, MERRF, Methylmalonic aciduria, MIDD, MLASA, MNGIE, NARP, NDFUF2, NDUFV2, NDUFAF1, PEO, Sengers Syndrome, TMEM70 deficiency, SANDO, SCAE
DCM	CI and cytochrome C oxidase deficiencies, CII deficiency, DCMA, Kearns-Sayre syndrome, Leigh syndrome, LHON, MELAS, MERRF, Methylmalonic aciduria, MLASA, MIDD, PEO, Propionic aciduria, SANDO, SCAE
LVNC	Barth syndrome, mutations in C1QBP, Combined methylmalonic aciduria and homocystinuria, DCMA, LHON
RCM	MIDD
HICM	CI deficiencies
Conduction defects/Arrhythmias	CI deficiencies, CPEO, KSS, LHON, MELAS, MERRF, MNGIE, MLASA, PEO, PRKAG2-related disease, SANDO, SCAE, TMEM70 deficiency

CI, complex I; CII, complex II; CPEO, Chronic Progressive External Ophthalmoplegia; DCM, dilated cardiomyopathy, DCMA, dilated cardiomyopathy with ataxia; FRDA, Friedreich's Ataxia; HCM, hypertrophic cardiomyopathy, HICM, histiocytoid cardiomyopathy; LHON, Leber hereditary optic neuropathy; LVNC, left ventricular non compaction; MELAS, melas mitochondrial encephalomyopathy lactic acidosis and stroke like episodes; MERRF, Myoclonic Epilepsy with Ragged Red Fibers; MIDD, Maternally Inherited Diabetes and Deafness; MLASA, Mitochondrial myopathy, lactic acidosis and sideroblastic anemia; MNGIE, Mitochondrial neurogastrointestinal encephalopathy syndrome; NARP, Neuropathy, ataxia, and retinitis pigmentosa; PEO, progressive external ophthalmoplegia; SANDO, sensory ataxic neuropathy, dysarthria and ophthalmoparesis; SCAE, spinocerebellar ataxia with epilepsy.

Imaging studies and others instrumental evaluations should be considered following the clinical and diagnostic clue. Abdominal ultrasound, fundoscopic evaluation, hearing evaluation, brain magnetic resonance image (MRI) with spectroscopy and spinal MRI, neurophysiological studies with visual, auditory, somato-sensory evoked potentials, electromyography and nerve conduction studies are part of the multisystemic evaluation and screening in the suspicion of MD. Cardiac evaluation with echocardiography, ECG studies and 24h-ECG monitoring are pivotal in the diagnosis and follow-up of suspected MD with cardiac involvement.

Further investigations are more specific when suspecting a MD and useful to guide the further molecular investigations. Recently, consensus statements on the diagnosis and managements of MD have been provided (92). In case of suspicion of MD, a fresh skeletal muscle biopsy is considered the gold standard in the diagnosis. Therefore, a functional assay on skeletal muscle to measure the RC complex activity, using a spectrophotometric method, and to determine, though histochemical staining, histological and histochemical analysis to possibly identified a distinctive pattern as the typical ragged-red fibers (RRF), by using the modified Gomori trichrome stains or the altered complex IV activity with dual COX/SDH stain, in the so called “Cox negative fibers”, is highly recommended. An immunofluorescent technology using labelled antibodies against subunits of complex I-IV has been recently introduced and allows a precise quantification of RC deficiency of all different complexes. Moreover, analysis by electron microscopy examination of muscle tissue can allow the identification of mitochondrial proliferation and abnormal morphology in mitochondrial myopathy (93).

Mitochondrial function can also be assessed though simultaneously measure of extracellular flux analyzer evaluating the mitochondrial respiration and glycolysis and though analysis of mitochondrial dynamic (fusion-fission equilibrium).

Molecular testing includes the mt-DNA and the evaluation of mtDNA content. A reduced level of mtDNA content implies defects in mtDNA biosynthesis, leading to DNA depletion. The estimation of mtDNA copies is performed by real-time quantitative polymerase chain reaction using a mtDNA probe and unique nuclear gene reference. When a nuclear-encoded MD is suspected, a next-generation sequencing of known genes panel associated with MD is useful and cost effective. When the clinical picture is not suggestive of a specific gene or group of genes and a more extensive genetic investigations is needed, a whole exome or genome sequencing should be considered (94).

Natural history studies have demonstrated both the high prevalence of cardiac disease and the deleterious effects on patient outcome of a cardiac presentation. A significant difference in survival to age 16 years was noted in 113 children with MD (18% and 92%, respectively, in those with and without cardiomyopathy) (21). This result, in a cohort including patients with mtDNA and nDNA mutations, has subsequently been confirmed in other large paediatric cohorts (95, 96). Adult studies, in patients with mtDNA mutations exclusively, have established the progressive nature of cardiac involvement (10, 97, 98) with important impacts on morbidity and early mortality (99, 100). In common with many newly recognized disorders, early reports of cardiac involvement in mtDNA disease featured patients with severe phenotypes. Family genetic screening has undoubtedly broadened the spectrum of mtDNA disease to include more asymptomatic or oligosymptomatic adults, perhaps limiting the applicability of early studies. A recent study of 32 adult patients demonstrated that, although cardiac involvement was apparent in 78% patients, minor electrocardiogram (ECG) abnormalities represented the most common manifestation, with cardiomyopathy present in 25% patients (101). Progressive systolic dysfunction and high-grade AVB did occur in a minority, but the incidence of severe CV complications was relatively low over a median follow-up of 4 years. Large multi-centre prospective clinical cohort studies are underway and will provide novel insights into the natural history and response to intervention of adult mtDNA disease (102).

## 7 Therapeutic strategies for MCMPs

The early recognition of the MCMPs is essential for the correct general assessment and management.

Most of the therapeutic interventions are applied when patients are symptomatic and are supportive with the major aim to improve quality of life and life expectancy. These interventions are focused on management of nutritional aspect, respiratory disorders, neurological and muscular involvement, hearing and vision disorders, diabetes.

Although there is no strong evidence supporting the use of any specific therapy for the primary MD (103), several agents aiming to enhance mitochondrial function, to reduce the oxidative stress and to fill the shortfall in specific molecules are generally prescribed.

These treatments include cofactors of the mitochondrial enzymes (coenzyme Q10, idebenone, riboflavin, dichloroacetate, and thiamine), energy buffer (creatine), antioxidant agents (vitamin C, vitamin E, lipoic acid, cysteine donors, and EPI-743), amino acids restoring nitric oxide production (arginine and citrulline), cardiolipin protector (elamipretide), agents enhancing mitochondrial biogenesis (bezafibrate, epicatechin, and RTA 408), and nucleotide bypass therapy (104). Mitochondria-targeted therapies such as Elamipretide have recently emerged as a topic of scientific and clinical interest. This molecule may be promising in the clinical setting (105).

To date, gene therapy, which would offer the possibility of a specific therapy for each single mutation, is reserved for experimental protocols and currently remains a hope for the future (106).

### 7.1 Cardiovascular therapies and heart transplant

Patients with MCMPs have a very poor prognosis. Mortality up to 82% at 16 years of age has been reported, with CMP as the leading cause of death (11).

Therefore, every effort must be made to guarantee the best cardiovascular assistance to these patients. Ethical considerations should be paid during the decision process, taking in account the progression of the disease, with a sharing decision process with patients and caregiver.

In the absence of specific studies on cardiovascular therapy for these patients, the management of cardiomyopathy, HF, bradyarrhythmias and tachyarrhythmias, follows the same guidelines as those for the general population (107).

Obviously, the degree of multisystemic involvement and the expected quality of life must be carefully evaluated by an interdisciplinary team before proposing transplantation. Some conditions such as severe neurological involvement, expressed with repeated strokes or severe dementia, as well as significant muscle wasting and cachexia may, indeed, contraindicate transplantation (108).

To date, there are limited but encouraging data on heart transplant in this population. Bonnet et al. described successful heart transplantation for 7 patients with largely isolated MCMPs. Range of age was 1 month to 16 years and all had DCM with hypertrophic walls (109).

Case reports described successful heart transplantation also in adolescents with MCMPs and encephalopathy (108, 110).

Santorelli et al. reported a case of a 3 years old girl with a very rare mitochondrial depletion syndrome, who was successfully treated by heart transplantation for advanced dilatative phase of HCM. No severe complication after heart transplant nor significant extracardiac involvement were described during the 4 years follow-up post-transplantation (54).

Recently, Weiner et al. analysed data from US registries of 1,330 heart transplant paediatric recipients. They compared the outcomes and comorbidities of children with mitochondrial aetiology (47 cases, 3.5% of the total) to others without MD, demonstrating equivalent survival with less acute rejection in MD patients and no difference in infection or malignancy after heart transplant (111).

Patients with MD were more prone to stroke, required longer post-transplant intensive care assistance and longer duration of mechanical ventilation compared to those without it. The rate of hospital readmission in the first-year post-transplant was similar between the two groups albeit with longer hospitalization for children with MD. Moreover, over a median follow-up of 4 years, the survival rate was similar in the two groups.

In the cohort of MD patients, no significant extracardiac involvement was reported. The CMP phenotype was dilated in 83% of cases, hypertrophic in 13% of cases and 1 patient had LVNC and histiocytoid cardiomyopathy, while no RCM was reported (112).

In case of HF and hemodynamic instability despite maximal medical therapy, short- and long-term mechanical ventricular assist devices (VAD) should be considered. In the literature some cases of children and young adults with MD assisted with VAD are reported, both as destination therapy and as bridge to transplantation (113, 114).

### 7.2 Perioperative management

MD present unique challenges inpatient management. During hospitalization, especially during perioperative setting, a meticulous maintenance of normal glucose levels, oxygen balance and good gas exchange is necessary to minimize acidosis. Prolonged fasting must be avoided. 0.9% saline and 5% glucose should be administered to maintain an adequate blood sugar level, while intravenous solutions that contain lactate should be avoided, always respecting any water restriction in case of HF. Whereas hypothermia can worsen mitochondrial function, intravenous fluids are recommended to be warmed, and the use of thermal blankets should be considered (115). Moreover, special care is needed during general anaesthesia, because most of the in-use anaesthetic drugs can impair the mitochondrial metabolism. Although a strong association between MD and malignant hyperthermia has been dismissed, patients with myopathy should be considered at risk, thus the use of volatile anaesthetics and succinylcholine should be avoided (116–118). There could be also a high risk of aspiration due to oesophageal dysmotility and to central nervous system disorders, a high risk of seizures, coagulopathy and electrolyte abnormalities related to labile acid-base balance.

For all these reasons invasive monitoring during perioperative assistance should be strongly suggested (116).

Important considerations should be given to the management of post-transplant immunosuppressive therapy. Generally standard regimen including calcineurin inhibitors such as cyclosporine and FK506 is used. However, the use of these drugs in patients with MD has not been clearly defined. In detail, there is conflicting evidence regarding the potential effect of calcineurin inhibitors on oxidative stress, especially in MELAS. And this is the reason why someone suggests the substitution with a rapamycin-based regimen in this unique patient population (108).

## 8 Conclusions

MD are clinically heterogeneous and difficult to suspect and diagnose. The cardiac involvement in MD in children and adulthood encompasses a wide range of clinical presentations, from CMP to conduction abnormalities and can have a huge impact on prognosis. To date, however, the available evidences on cardiac involvement in patients with MD are still poor and elusive, and guidelines for clinical management and cardiovascular risk stratification remain an unmet need.
