# Supplementary figures and images for: Mitochondrial cardiomyopathies: navigating through different clinical and management pictures between adult and paediatric forms
Source: Front Cardiovasc Med. 2025 Jul 3;12:1621096. doi: 10.3389/fcvm.2025.1621096 (PMC12267293; doi:10.3389/fcvm.2025.1621096)

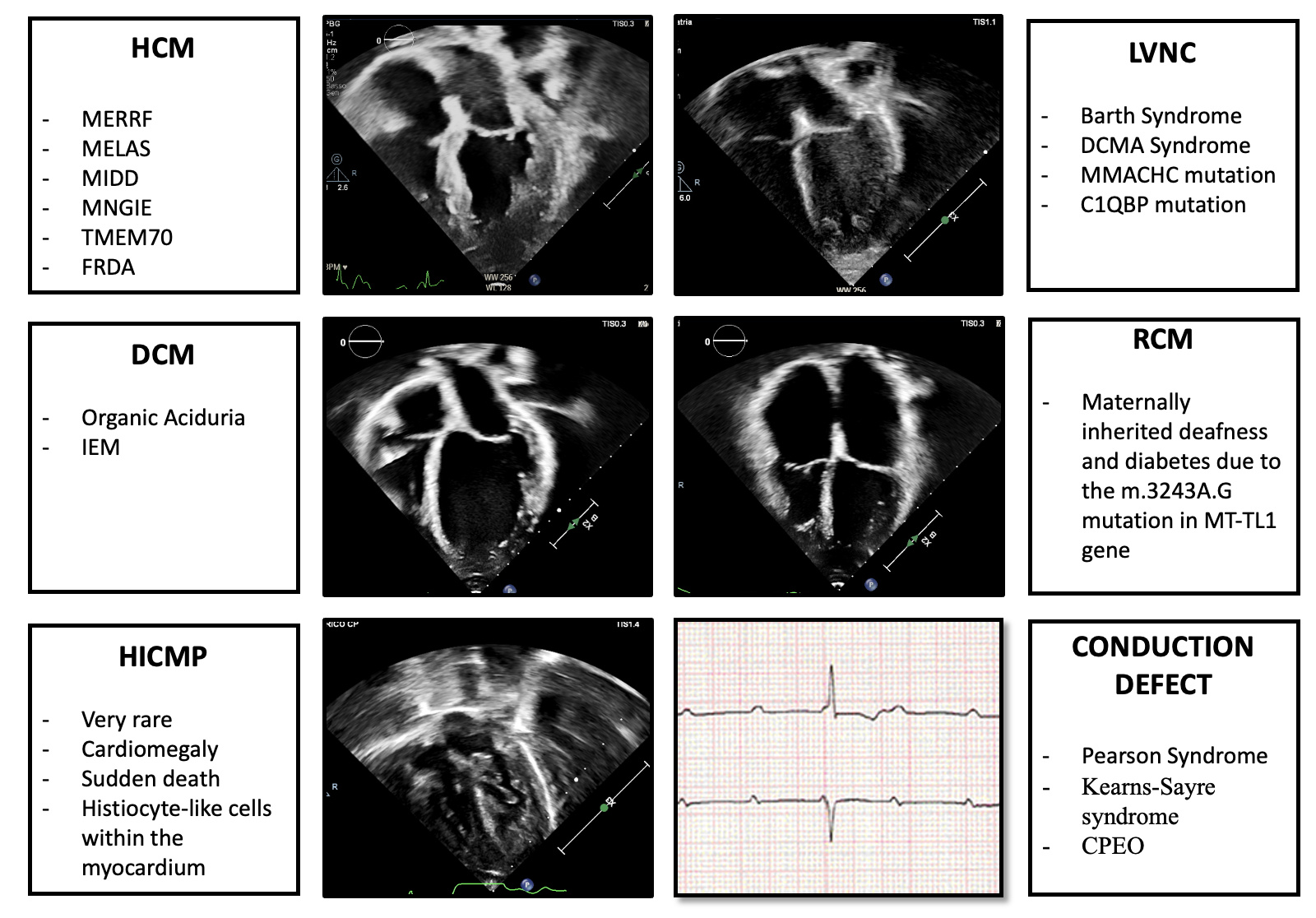

Supplement: Supplementary file 1 [file Image1.jpg]
